# Artificial intelligence will reduce the need for clinical medical physicists

**DOI:** 10.1002/acm2.12244

**Published:** 2018-01-15

**Authors:** Xiaoli Tang, Brian Wang, Yi Rong

**Affiliations:** ^1^ Department of Radiation Oncology Memorial Sloan Kettering Cancer Center West Harrison NY USA; ^2^ Department of Radiation Oncology University of Louisville Louisville KY USA; ^3^ Department of Radiation Oncology University of California Davis Comprehensive Cancer Center Sacramento CA USA

## 1 INTRODUCTION

In 2011, IBM's supercomputer Watson defeated the former human winners and won the first prize on *Jeopardy!* game. It has created an overly publicized attention on machine learning and Artificial Intelligence (AI). Early this year, Google AlphaGo has marked a major breakthrough in AI by winning the first game against the world's best champion human player in the world's most complex game, the ancient Chinese *Go* game. With no doubt, the interests in AI and its related products had reached a global frenzy. As scientists advance in technology, a concern of job security has risen up: will robots take our jobs? IBM Watson has evolved from a “question answering machine” to a highly intelligent “cognitive diagnostic engine” or a “decision support system” over the past 6 yr. Based on Carl Frey and his collaborators, future family health centers may transition to a team of nurse practitioners with the support of Watson Health and overseen by one single doctor.^1^ Will AI technology also marginalize medical physicists in the near future? In this series, we have Dr. Xiaoli Tang arguing for the proposition that “AI will reduce the need for clinical medical physicists” and Dr. Brian Wang arguing against it.

Dr. Xiaoli Tang received a Ph.D in Electrical Engineering from the Rensselaer Polytechnic Institute. She then did her postdoctoral training in Medical Physics at the Massachusetts General Hospital and the University of California at San Diego. She previously worked at the University of North Carolina and now is working as an Assistant Attending and chief physicist at the Memorial Sloan Kettering Cancer Center Westchester regional site. She is an expert in motion management, Deep Inspiration Breath Hold (DIBH) for left‐sided breast cancer, and machine learning algorithms on medical physic applications. She is interested in developing related clinical trials, and bringing new technology to the clinic. She is a member of the American Association of Physicists in Medicine (AAPM), and the American Society for Radiation Oncology.

Dr. Brian Wang received his PhD in nuclear engineering from Rensselaer Polytechnic Institute in Troy, NY in 2005. He currently works at University of Louisville as the chief of physics and medical physics residency director. Dr. Wang is an associate editor for the JACMP. His research interests include motion management, image guidance, and SRS/SBRT. Dr. Wang has been involved with the AAPM Spring Clinical Meeting and its predecessor ACMP annual meeting as a program director or the subcommittee chair for 8 yr. Dr. Wang serves on several committees at ASTRO, RSS, and ABR.

## 2 OPENING STATEMENTS

### 2.A Xiaoli Tang, Ph.D

Technology always puts existing jobs under strain. Without doubt, the next technological evolution is the Artificial Intelligence (AI). Already, there is an estimate of 20–40 M jobs in peril in the US from developments in AI and its related technology, which counts 15%–30% of the US labor force.^2^ This is happening in clinical Medical Physics as well. Out of many duties that medical physicists have taken upon, the clinical aspect mainly includes treatment planning, chart checking, and machine quality assurance (QA). The need of clinical physicists in these areas has already been slowly reduced over the past several years, and this speed is going to be increased as more AI technologies are implemented in the clinic.

Let us look at therapeutic physics first. The need for physicists on planning has largely been shifted to dosimetrists. With the AI‐fueled automatic planning software, the need for physicists will be further reduced or eventually eliminated. Many articles have been published on knowledge‐based planning.^3^, ^4^, ^5^, ^6^ It is a novel treatment planning technique capable of estimating the dose volume histograms (DVHs) of organs at risk based on the DVHs of previous plans with similar characteristics. Vendors have implemented this to their new automated or semi‐automated treatment systems. Varian's (Varian Medical Systems Inc, Palo Alto, CA, USA) RapidPlan allows a clinic to use their own database of plans to create a knowledge‐based RapidPlan model for a given site. This model can then be applied on new cases to generate plans. Philips’ (Philips HealthCare, Stamford, CT, USA) Pinnacle Auto‐Planning takes a different approach. It allows users to state the dosimetric planning goals and then automatically creates planning objectives and tuning structure iteratively to meet the goals. RaySearch (RaySearch Laboratories, Garden City, NY, USA) also has its automated planning module. One of its successful applications is the rayAutoBreast. It is a fully automated IMRT treatment planning solution for tangential breast, including segmentation of all relevant structures, beam placements, setting optimization parameters, calculating dose, and even plan reporting.

Research has shown that on average, these automatic planning systems can achieve planning target volume (PTV) coverage that is highly comparable with the original plan. The normal tissue sparing is also within acceptable range.^7^, ^8^ The performance of these commercial systems is expected to further improve in the following years, and these systems will gradually replace the manual planning process, at least for those standard plans. It seems inevitably that the need of routine planning by physicists or dosimetrists is diminishing by then. Some clinics have already started planning on letting dosimetrists do the initial chart checksum the automatically generated plans, where the initial chart checks are now routinely performed by physicists.

Initial chart check is a thorough checking process after the plan is approved and finalized. This is the second area that the need of physicists will be reduced. One source of planning problems is contour discrepancy and/or interuser variations. Machine learning is an application of AI. Currently, the machine learning‐based autosegmentation systems can reliably contour structures with standard shapes or can be distinguished out from surroundings, such as bladder, rectum, heart, lungs, etc. With the improvement of AI, segmentation applications might be able to contour more challenging structures, such as prostate, spinal canal, etc. There have been many studies on the diagnosis area on automatic tumor segmentation.^9^, ^10^ In the therapeutic area, AI‐based systems might later be able to contour PTVs as well. These new technologies will reduce potential variations or inaccuracy of contours and lead to less chart checking problems. Consequently, the need for physicists will be reduced as well.

Machine QA is the third area that will need less physicist efforts. There has been research on predicting machine output trends using AI‐based algorithms.^11^ The proposed data visualization can predict the Linac performance over time and prompt physicists to perform output calibration before the output is drifted away from the tolerance. The routine Linac output checks (daily, monthly or annual) are currently recommended as a standard. Yet, based on our experience, modern linacs are getting so stable that we might not need to routinely perform monthly output calibration. In our clinic, output calibration happens on average once per 6 months. This suggests that , with AI‐based predictions, we perhaps no longer need to check machine output on a monthly basis. Similarly, data visualization is also an effective tool to perform data comparisons, alert failures, and potential identify causalities. All these may lead to the reduced frequency of medical physicist interaction.

For diagnostic physicists, the need for medical physicists may also be reducing. One of the major responsibilities is the optimization of the clinical imaging procedures.^12^ Some vendors have already automated this process through implementing AI‐based solutions. For instance, IBM Watson is able to review a digital chest x ray and suggest that the patient may have small‐cell lung cancer and heart surgery. Watson can then go ahead and search PACS, EMR, and departmental reporting system to bring in related files without any physics interaction.^13^ Similar to therapeutic physic situation, the need for physicists’ machine QAs might be reduced due to the AI‐based predictions of the machine performance.

### 2.B Brian Wang, Ph.D

Clinical physicists in radiation oncology engage in two general types of work: routine and nonroutine activities. Routine work includes scheduled machine QA, weekly chart checks, initial chart check, and review before first treatments and brachytherapy procedures. AI can surely facilitate chart checks, but it cannot reduce the time when physicists need to communicate with therapists and dosimetrists for potential issues. As for brachytherapy procedures, most of the physics efforts is operational and cannot be reduced by an AI technology.

Examples of nonroutine activities are commissioning treatment machines, implementing new techniques into clinical practice and providing patient‐specific consultations. Most of these efforts require constant interactions with other team members and cannot be easily reduced by AI technologies. If you ask clinical physicists what they have done at the end of a day, they usually cannot recall the list of tasks despite of a hectic clinical day. They are constantly pulled away by other team members for professional consultations. As technologies and treatment options get more complex in healthcare, clinical medical physicists will get involved in more nonroutine activities, which cannot be reduced by AI technologies.

A recent publication advocates for more direct patient interactions by clinical physicists in the future and I full‐heartedly agree with the authors for their following remarks.^14^ “The future clinical medical physicist should routinely meet with patients during their course of treatment to educate them on the intricacies of their treatment plan and delivery, and answer any questions. Eventually, functions such as determining optimal image combinations, target volume delineation, and shared decision making with the radiation oncologist should be expected of a clinical medical physicist.” The current responsibilities of clinical physicists have already changed drastically from those in the 1990s. They will keep adapting to those roles as described by this paper in the future. When this happens, it is hard to imagine how an AI machine can meet with patients and discuss their treatment plans.

Now let us look at diagnostic physicists, who account for about 20%–30% of the AAPM membership. Currently, many of the clinical physics tasks are performed by radiation technologists and only those regulatory‐required procedures are done by clinical medical physicists. In recent years, more diagnostic physicist positions have been posted at the AAPM placement center. This is a result that clinical physicists have demonstrated their value in the patient care process such as imaging protocol design. Administrators and radiologists start realizing the importance of clinical medical physicists and now need to recruit more. As AI technologies are increasingly used in radiology department, more clinical physicists are needed to implement them for clinical use.

It is easier for AI to master tasks requiring less intelligence. Indeed, autonomous cars with AI technology will probably dominate the roads in the near future, but medical physicists engage much more complex work and require specialized training postgraduate education. Almost anyone can drive a car after attending a driving school, but clinical physicists need to accumulate years of experience to solve problems, which are difficult for AI to master. In addition, QA of an AI system would be very challenging and it demands more physicists’ work. If an AI technology was treated as a black box, a catastrophic damage may occur. QA and commissioning of an AI technology will require tremendous time and effort by clinical medical physicists.

From 1997 to 2017, the total number of AAPM members has been more than doubled from 4327 to 8786 (Private communication with AAPM Membership Committee Staff Jennifer Hudson). Let us assume the percentage of clinical medical physicists stay the same within the AAPM membership. We can then infer that the number of clinical medical physicists has doubled over the last 20 yr. This is because more clinical physicists are needed as technologies become more complex in both radiation oncology and radiology. Even though the concept of AI did not exist during this period of time, utilization of computer‐assisted automation has definitely grown, from trending QA activities to knowledge‐based planning. Based on this trajectory, the number of clinical medical physicists will probably double in 20 yr especially with AI being used more clinically.

## 3 REBUTTAL

### 3.A Xiaoli Tang, Ph.D

“AI can surely facilitate chart checks, but it cannot reduce the time when physicists need to communicate with therapists and dosimetrists for potential issues.” It looks like that my opponent also agrees that AI can facilitate chart checks. As mentioned in the open statement, with the automatic segmentation and planning, it is fair to say that AI can reduce the burden of chart checking through reducing interuser variations. This is not entirely eliminating the need of communication with therapists and dosimetrists if we saw issues during chart checks. Yet, there is no doubt that the less issues we find in chart checks, the less amount of work and time we need to devote to communicating with therapists and dosimetrists. In other words, there will be less need for clinical physicists.

It is true that technologies and treatment options are getting more complex in healthcare. However, higher complexity does not necessarily mean more work. One good example is the Varian TrueBeam. Comparing to all Varian's previous models, TrueBeam is much more advanced in design and complex in functionalities. Yet, based on my personal experience, it is more reliable and stable, thus requires less clinical physics efforts. Currently, TrueBeam, or any other commercial Linac on the market, only has the capability to alert users if any machine issues occur. In the foreseeable future, with enough data collected, these Linacs can evolve to having the capability of predicting certain machine behavior, which might lead to a need‐based machine QA with lower frequency. Similarly, the need to perform all routine QA items for commissioning will be reduced as well.

I echo my opponent that AI cannot replace human touch. In my mind, human touch is the most important element of patient care. A significant benefit of having AI is to increase our time in interacting with patients, as AI will reduce routine clinical workload for medical physicists. One example is IBM Watson Health, which is a pioneer AI application in health care. They can “develop a more individualized, patient‐centric approach to oncology while helping to increase time for patient‐physician interactions^13^”, as mentioned in the last paragraph of my opening statement.

Regarding the statement that it is easier to master less intelligent tasks like driving for AI, I would disagree. Current AI technology can already automatically identify human anatomy and perform contour segmentation. These tasks are traditionally performed by MDs and/or dosimetrists, who acquire the skills through years of professional training. Other intelligent tasks that AI has proven capable of in our field include autoplanning and autochart checking. With no doubt, AI is progressing in a direction to not only master what humans can do but also trump us.

In the last paragraph, my opponent has observed that the number of AAPM members has doubled in the past 20 yr. While the number of new cancer cases has increased from 1.3 million in 1997 to 1.7 million in 2017 based on www.cancer.gov, it is not proportional to the increased number of medical physicists. I would agree that this might due to the increase in complex technologies, that is, multileaf collimator (MLC) and intensity‐modulated radiotherapy (IMRT). However, AI is still at its infancy stage. We know that usually it requires more efforts when we adopt new technologies. What clinical medical physicists would do is more QA at the beginning. It is very likely that we see an initial increase in the need of physicists for QA and clinical implementation. However, eventually, as much as we do not want to admit, the need of clinical medical physicists in cancer care will be gradually reducing as the AI technology matures.

### 3.B Brian Wang, Ph.D

For radiation therapy, Dr. Tang listed three areas that AI could reduce the need of clinical medical physicists: treatment planning, plan checking, and machine QA, which all fall into the routine activities. While I agree that AI technology may help clinical medical physicists spend less time on these types of activities, I disagree that AI would reduce the need of clinical physicists. First, all these AI technologies require carefully commissioning into clinical practice. For example, it is not a trivial job to develop a knowledge‐based planning model that is customized to someone's own institution. Second, as also noted by Dr. Tang, the treatment plans generated by AI have to be extensively checked by a human, either a dosimetrist or a physicist. Last but not the least, it creates a new challenge and task to implement routine QA procedures for these AI technologies. Currently, most of the research and development effort for AI technologies stems from industries and academic institutions. As AI starts migrating into clinical practice, the associated supporting resource will shift to the hands of clinical physicists.

As for diagnostic imaging, the current clinical physicists are already facing a specialty identity issue. While medical physicists continue making important contributions to the field, their clinical roles have not always been viewed as critical.^15^ With AI being utilized more in the field, additional efforts are needed by clinical medical physicists to understand an imaging artifact, to optimize a scan protocol or to monitor an equipment performance. Most of these tasks cannot be generalized and have to be customized to each individual institution and patient group.

For both diagnostic imaging and radiation therapy, the values of clinical physicists are demonstrated not only by routine QA activities, but more importantly by nonroutine activities. Commissioning a new technology for radiation therapy is not as simple as measuring LINAC dosimetric performance anymore. It involves clinical physicists to communicate with all team members within the department. Similarly, setting up a new imaging protocol is not just creating a set of parameters; instead, it requires clinical physicists to discuss the process with technologists. Quite frequently, clinical medical physicists need to provide in‐service education to other team members annually or before the initial implementation. A couple decades ago, clinical physicists may be seen as the “phantom” of the department: they do all their work such as patient and machine QA at nights and on the weekends. Now, clinical physicists have already become an indispensable team for patient care, especially in radiation therapy. In the future, clinical physicists will get involved in more patient‐specific consultations, which is very difficult to be replaced by an AI machine. Now at the organizational level, AAPM has initiated the Medical Physics 3.0 project to redefine and reinvigorate the role of physics in modern medicine. With the growing public and collegial awareness of our role, the demand for clinical medical physicists can only increase as technologies such as AI are becoming more complex in healthcare.

## CONFLICT OF INTEREST

The authors declare no conflicts of interest.
